# Semantic representation of monogenean haptoral Bar image annotation

**DOI:** 10.1186/1471-2105-14-48

**Published:** 2013-02-12

**Authors:** Arpah Abu, Lim Lee Hong Susan, Amandeep Singh Sidhu, Sarinder Kaur Dhillon

**Affiliations:** 1 Institute of Biological Sciences, Faculty of Science, University of Malaya, 50603, Kuala Lumpur, Malaysia; 2 Curtin Sarawak Research Institute, Curtin University, Sarawak, Malaysia; 3 School of Biomedical Sciences, Faculty of Health Sciences, Curtin University, Perth, Australia

## Abstract

**Background:**

Digitised monogenean images are usually stored in file system directories in an unstructured manner. In this paper we propose a semantic representation of these images in the form of a Monogenean Haptoral Bar Image (MHBI) ontology, which are annotated with taxonomic classification, diagnostic hard part and image properties. The data we used are basically of the monogenean species found in fish, thus we built a simple Fish ontology to demonstrate how the host (fish) ontology can be linked to the MHBI ontology. This will enable linking of information from the monogenean ontology to the host species found in the fish ontology without changing the underlying schema for either of the ontologies.

**Results:**

In this paper, we utilized the Taxonomic Data Working Group Life Sciences Identifier (TDWG LSID) vocabulary to represent our data and defined a new vocabulary which is specific for annotating monogenean haptoral bar images to develop the MHBI ontology and a merged MHBI-Fish ontologies. These ontologies are successfully evaluated using five criteria which are clarity, coherence, extendibility, ontology commitment and encoding bias.

**Conclusions:**

In this paper, we show that unstructured data can be represented in a structured form using semantics. In the process, we have come up with a new vocabulary for annotating the monogenean images with textual information. The proposed monogenean image ontology will form the basis of a monogenean knowledge base to assist researchers in retrieving information for their analysis.

## Background

Over the years, we have been collating information on monogeneans found in Malaysian waters, digitized these images and stored them as unstructured data. These images which were extracted from journal publications are meaningless without their textual annotations. Contemporary approaches to organizing image data and its corresponding textual descriptions are by using either the relational database technologies or the XML technologies. For example, the Biota [1], InsideWood [2], MonoDb [3] used the relational database technology, while the Open Microscopy Environment (OME) Data Model and XML File [4], knowledge-based grid services for high-throughput biological imaging [5], PLAZi [6] utilised the XML technology. Annotations of images in a relational database are confined by the number of columns used for the descriptions of the images. The number of characters allowed in a cell of a database table is also fixed. Any new inclusions into existing relational model with fixed tables and set of fields may require new schema to be developed and existing queries to be revised. Migration to a new schema and revision of queries can be very cumbersome and time consuming. Excessive images stored in a database take up a lot of space and creates a huge database file, affecting retrieval time. Storing images outside the database file in a directory and linking them via identifiers in the database column was a possible solution but here again any new inclusion of data will required a change in identifiers [7,8]. XML is normally used to describe and structure data [9]. Annotations of images in XML are not linked and hence the relationships between objects are not expressed.

Semantics is needed to organize data by focusing on the meaning of objects by expressing relationships. It provides necessary vocabularies to link different data entities to their properties [10].

**Figure 1:**
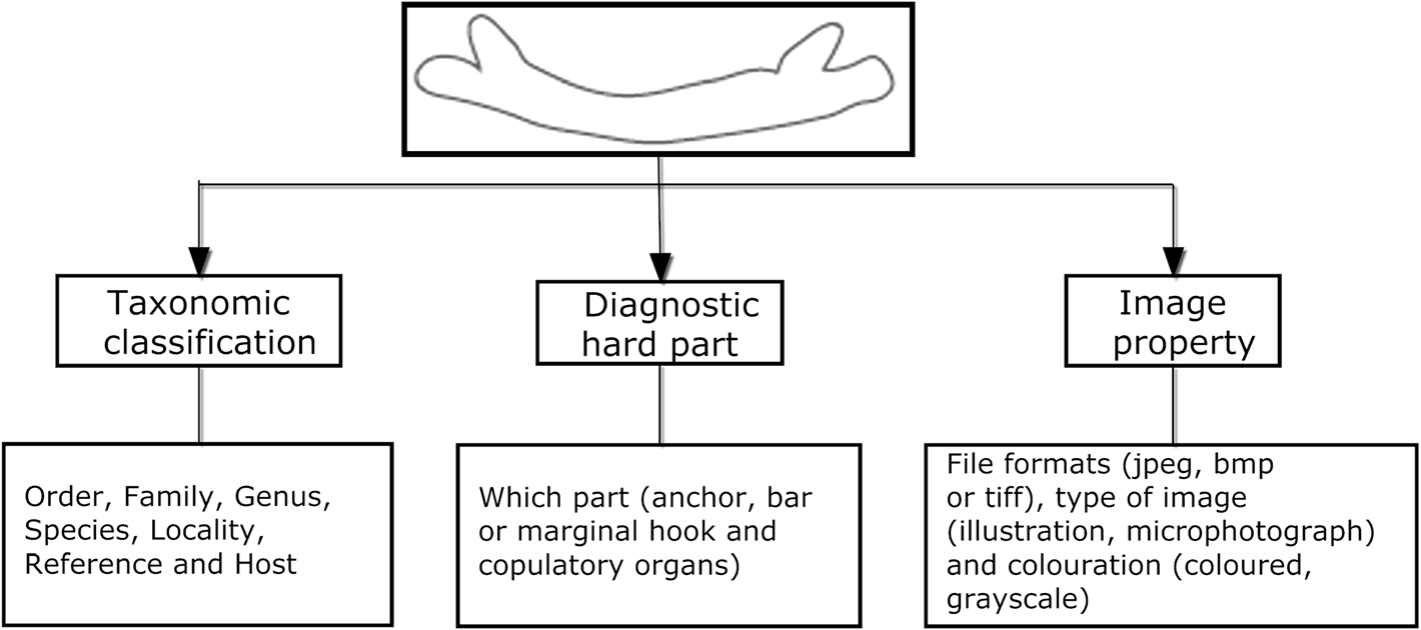
**Image is annotated with textual information.** An image of haptoral bar of the monogenean species is annotated using relevant textual information.

In this paper, we intend to semantically annotate the haptoral bars of the monogenean species in a structured manner with their textual information or descriptions (see Figure 1) for retrieval purposes.

## Methods

### Identifying concepts

Data used in this paper are images of the monogenean haptoral bars along with textual information which consist of: (1) taxonomic classification and (2) description of properties of an image found in publications (see Figure 1). The data is analysed and structured into main concepts. Defining these concepts using a standard structured vocabulary is necessary to make sure the meaning of data is clear and explicit, thus facilitating data sharing and maximizing reusability in wide variety of contexts.

The Taxonomic Data Working Group (TDWG) [11] strongly suggests the deployment of Life Science Identifiers (LSID), the preferred Globally Unique Identifier technology and transitioning to RDF encoded metadata as defined by a set of simple vocabularies. The TDWG LSID vocabulary has been widely used in biodiversity and offers a wide coverage of concepts, which are suitable to annotate the taxonomic information of an organism. The nomenclature used in this research is from TDWG LSID vocabulary and where necessary, appropriate vocabularies specific to the monogeneans are formed (see Additional file 1). Specific vocabularies (for example *DiagnosticPartTerms*) are needed as Monogeneans are parasitic platyhelminths and are distinguished based on both soft reproductive anatomical features as well as shapes and sizes of sclerotised hard parts such as the haptoral bar, anchor, hook and male and female copulatory organ [12].

Seven concepts are described from the monogenean data used in this paper - *Specimen, TaxonName, PublicationCitation, KindofSpecimenTerm, TaxonRankTerms, PublicationTypeTerms* are defined using the TDWG LSID controlled vocabulary, whereas the *DiagnosticPartTerm* is a new concept. *Specimen* concept represents the illustrated images of the haptoral bars of the monogeneans. *TaxonName* represents a single scientific name. *PublicationCitation* represents a reference to the publication of the monogenean species. *KindofSpecimenTerm* represents the specimen terms such as illustration, digital object and still image. *TaxonRankTerms* represents the taxon rank terms for taxonomic classification. *PublicationTypeTerms* represents type of publication for example an article in journal or book. *DiagnosticPartTerms* represents the name of the monogenean hard parts.

### Defining properties and relationships

There are two types of properties for the semantics representation which are object properties and datatype properties. Object properties are relationships between two individuals (link an individual to an individual), whereas datatype properties describe relationships between an individual and data values. The properties defined for the seven concepts are mentioned here and descriptions are available in Additional file 1.

#### Properties for specimen concept

Four object properties are defined under the Specimen concept; *kindOfSpecimen, isHaptorBar, isCitedIn* , *typeForName* and three datatype properties; *specimenId, imgDir* and *imgDescription*.

#### Properties for taxon name concept

Eight object properties are defined under the TaxonName concept; *rank, isBelong,part,hasSpecies,hasGenus,hasFamily,hasOrder,isHostedIn* and four datatype properties; *nameComplete, authorship, year* and *locality*.

#### Properties for publication citation concept

Two object properties are defined under PublicationCitation concept; *pubType* and *lists* and five datatype properties; *author, year, title, parentPublicationString, number*.

#### Properties for diagnostic part terms, kind of specimen terms, taxon rank terms, publication type terms concepts

One datatype property is defined for *DiagnosticPartTerms, KindofSpecimenTerms, TaxonRankTerms, PublicationTypeTerms* concepts, which is called *definedTerm*. This property is given a generic name as it will be used to bind multiple concepts together.

### Semantic representation of data using the Web ontology language

7 concepts, 27 properties, and the relationships between them represent conceptualization of the data used in this paper. This conceptual framework needs to be converted in a machine readable formal specification to reason about the identified concepts and eventually describe the data. This formal specification of shared conceptualization is called ontology [13].

**Figure 2:**
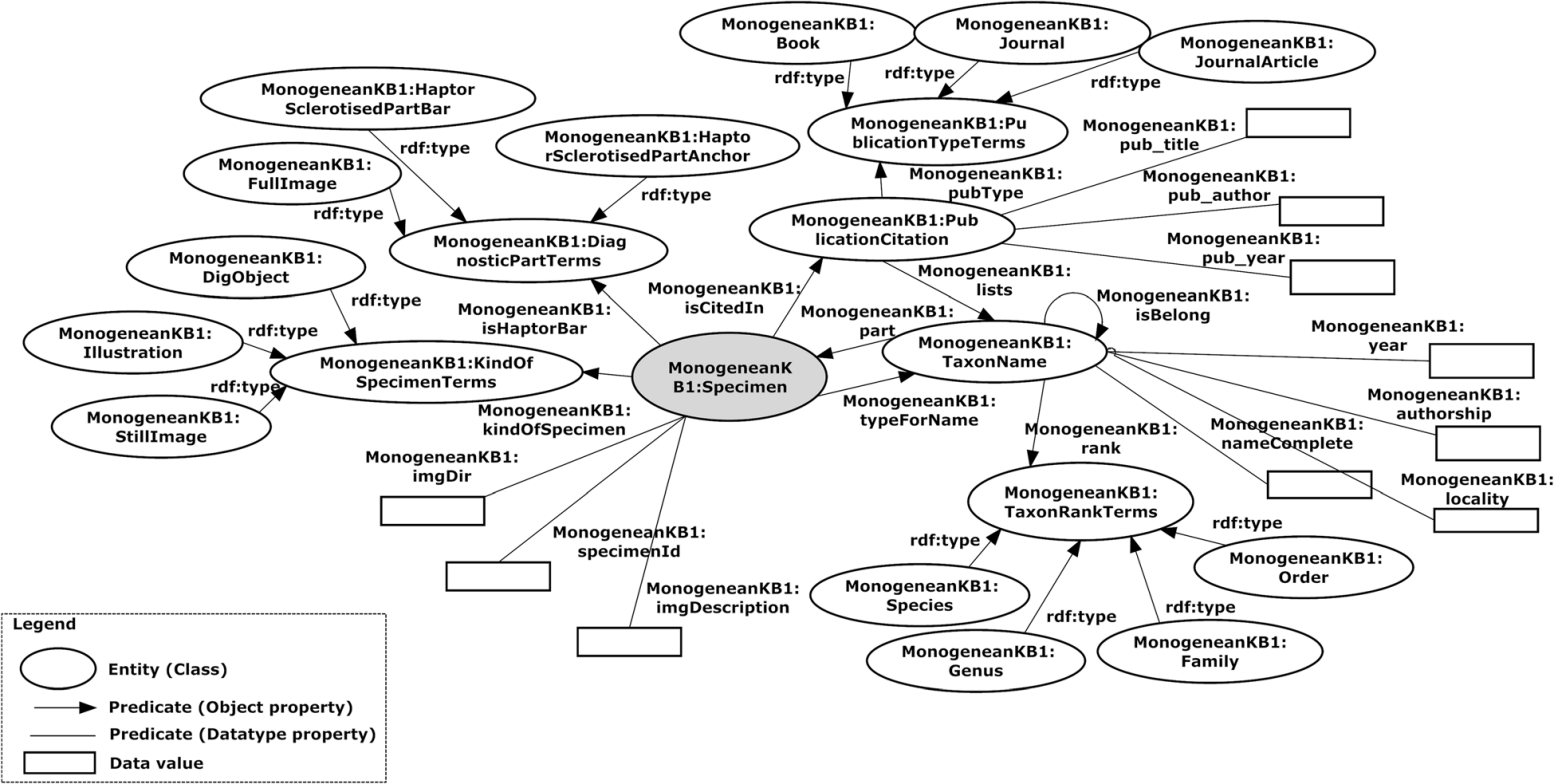
**A graph representation of triple statements.** Graph representation of multiple triple statements (the ovals represent the entities, the squares represent the data values in the specific entity and the lines represent the properties. Lines, the line with arrow heads and solid lines are directed from the subject (entity) to the object (entity or data value).

OWL [14] is an ontology language for the Semantic Web, developed by the World Wide Web Consortium (W3C) Web Ontology Working Group. OWL was primarily designed to represent information about categories of objects and how objects are interrelated—the sort of information that is often found in ontology. OWL can also represent information about the objects themselves—the sort of information that is often thought of as data [15]. OWL facilitates greater machine interpretability of Web content than that supported by underlying XML, RDF, and RDF Schema representations by providing additional vocabulary along with a formal semantics. In this paper we utilise ontologies in OWL format to represent a shared structured vocabulary that describe the monogeneans image data through the concepts, properties and relationships discussed above. Figure 2 depicts the whole ontology in a graph format.

### Data annotation

The data described by concepts is annotated in the form of *instance*s. While there are no fixed rules to name the *instance*s nevertheless the names should be reflective of the data they represent. For example, for the Specimen concept the record of each image of the haptoral bar or *instance* is given a unique label that will include its taxon name, diagnostic part depicted by the image and its sequence number in the directory (as shown in Table 1). There are 159 *instance*s for the *Specimen* concept*,* which represents all the haptoral bars of the monogenean images (see Table 2).

**Table 1:** **Naming of *instance *and number of *instance*s for each *concept***

***Concept***	**Naming of *instance***	**Name of *instance *(in bold)**	**Number of *instance*s**
TaxonName	*Instance* for species is named according to genus and species name	*instance* of species *Bifurcohaptor baungi* is labelled as **BifBaungi**	591
	The full name of genus is used for naming the genus *instance* name	*instance* of genus Bifurcohaptor is labelled as **Bifurcohaptor**	122
	The full name of family is used for naming the family *instance* name	*instance* of family Ancylodiscoididae is labelled as **Ancylodiscoididae**	35
	The full name of order is used for naming the order *instance* name	*instance* of order Dactylogyridea is labelled as **Dactylogyridea**	10
PublicationCitation	*Instance* for publication is named according to author and year	*instance* of publication Lim, L. H. S. & Furtado, J. I. (1983). Ancylodiscoidins (Monogenea: Dactylogyridae) from two freshwater fish species of Peninsular Malaysia. Folia Parasitologica. 30, 377 – 380 is labelled as **LimFurtado1983**	57
DiagnosticPartTerms	The full name of diagnostic part is used for naming the *instance*	*instance* of haptor sclerotised parts bar is labelled as **HaptorSclerotisedpartsBar**	3
KindOfSpecimenTerms	The full name is used for naming the *instance*	*instance* of illustration is labelled as **Illustration**	3
TaxonRankTerms	The full name is used for naming the *instance*	*instance* of species is labelled as **Species**	4
PublicationTypeTerms	The name of publication type is used for naming the *instance*	*instance* of journal article is labelled as **JournalArticle**	4

The naming of instances and number of instances of all the concepts.

**Table 2:** Concepts, instances, object or data type properties

***Concepts***	***Instance*s**	***Object properties***	***Datatype properties***	**Example of data**
Specimen	bif-baungi-vb-i1	kindOfSpecimen		Illustration
		isHaptorBar		Haptor Sclerotised parts Bar
		typeForName		BifBaungi
		isCitedIn		LimFurtado1983
			specimenId	j1-bif-bau-ven-bar
			imgDir	/images/BIF-BAUNGI-ventral-bar-single.jpg
TaxonName	BifBaungi	Part		bif-baungi-vb-i1
		rank		Species
		isBelong		Bifurcohaptor
		isHostedIn		SilBagMysHemurus
			nameComplete	*Bifurcohaptor baungi*
			authorship	Lim & Furtado
			year	1983
			locality	Tasek Bera, Pahang; Bukit Merah Reservoir, Perak
	Bifurcohaptor	Rank		Genus
		isBelong		Ancylodiscoididae
		hasSpecies		BifBaungi, BifIndicus
			nameComplete	Bifurcohaptor
			authorship	Jain
			year	1958

Example of concepts, instances, object or data type properties.

### Linking data from other ontologies

**Figure 3:**
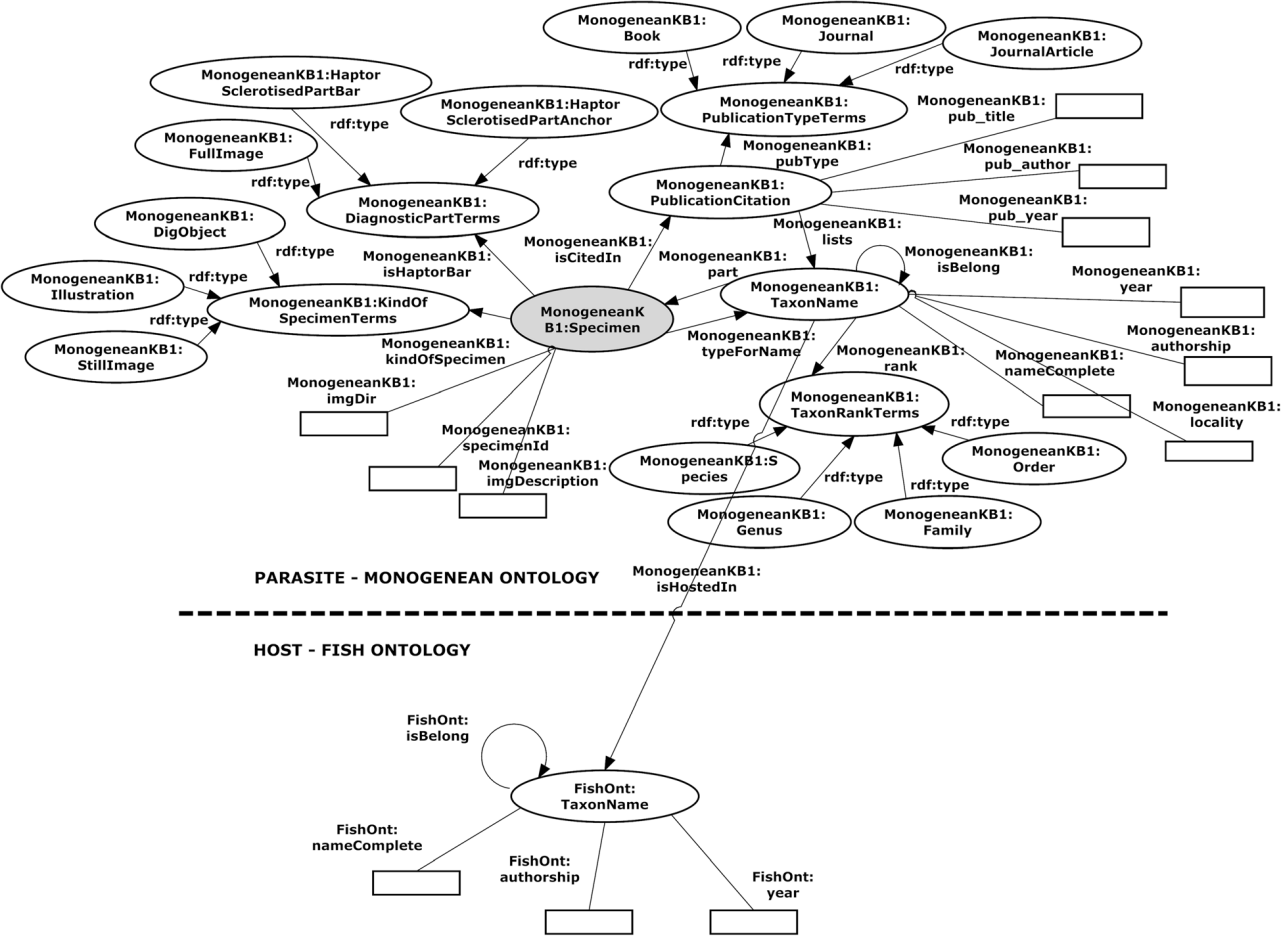
**A graph representation to demonstrate how the MHBI ontology is linked with the Fish ontology.** The monogenean TaxonName concept from MHBI ontology is linked with the fish TaxonName concept from the Fish ontology using the *isHostedin* property.

Since monogeneans species are parasites on fish, frogs and turtles, linking the monogenean data to their host data will provide more information about the monogeneans. In this paper, the data we used are basically of the monogenean species found in fish thus we decided to build a simple Fish ontology with *TaxonName* concept to demonstrate how the host ontology can be linked to the MHBI ontology. The two ontologies are merged by redefining the datatype property *(isHostedin)* in the *TaxonName* concept in the MHBI ontology as an object property to merge with the *TaxonName* concept in the Fish ontology as shown in the graph model (Figure 3).

### Evaluation

We may consider the ontology evaluation process either from the technical point of view (quality of the designed ontology), or from the practical view (usability of the designed ontology). For the purpose of evaluation of quality of the designed ontologies, we adopted five criteria suggested by Gruber [13] against which these ontologies will be evaluated. This methodology was successfully used previously to evaluate the Protein Ontology [16]. The five criteria are *clarity, coherence, extendibility, ontology commitment* and *encoding bias.* A discussion on how these criteria are applied to the concepts and properties in MHBI ontology is presented in the Results section.

## Results

### Evaluation

We introduce some level of formality into this discussion by adopting criteria suggested by Gruber [13] against which the ontology needs to be evaluated.

#### Clarity

Definitions within an ontology need to be stated in such a way that the number of possible interpretations of a concept would be restricted. This will contribute to the effectiveness of communication between agents. In the design of our MHBI Ontology, we stated that for each concept c with property p; the pair (c, p) exactly specifies a unique pair. During the design of MHBI Ontology this rule is enforced, and the uniqueness of the definition of concepts is guaranteed (see Figure 2). Clarity of MHBI Ontology is also checked by running 8 tests listed below and making sure all of them return true:

1. No Cardinality Restriction on Transitive Properties

2. No Classes or Properties in Enumerations

3. No Import of System Ontologies

4. No Meta-Class

5. No Properties with Class as Range

6. No Sub Classes of RDF Classes

7. No Super or Sub Properties of Annotation Properties

8. Transitive Properties cannot be Functional

**Figure 4:**
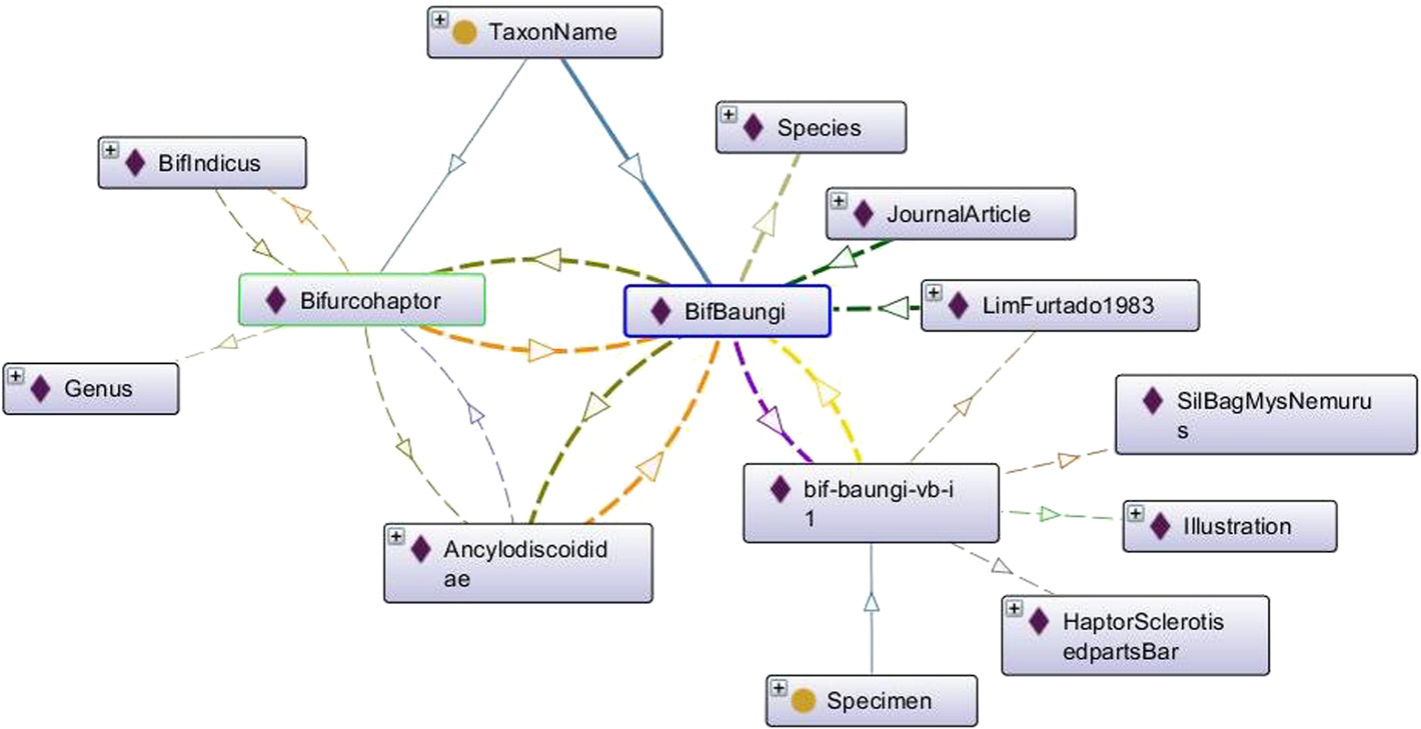
**Results of the Clarity criteria evaluation (Test 1 and Test 8); and the Coherence criteria evaluation (Test 6, Test 7 and Test 11).** An example of Transitive property for the clarity criteria test; and Functional, Inverse Functional and Inverse Transitive Functional properties for the coherence criteria test.

Example of result for Test 1 and Test 8 are as shown in Figure 4. Biological data is evolving over time whereby a new data type may need to insert into the ontology at any time. Thus for transitive properties we have not assigned any cardinality restriction. Besides that, it cannot be functional because it relates to more than one instance via the property. The example is explained further in Coherence Test 11.

**Figure 5:**
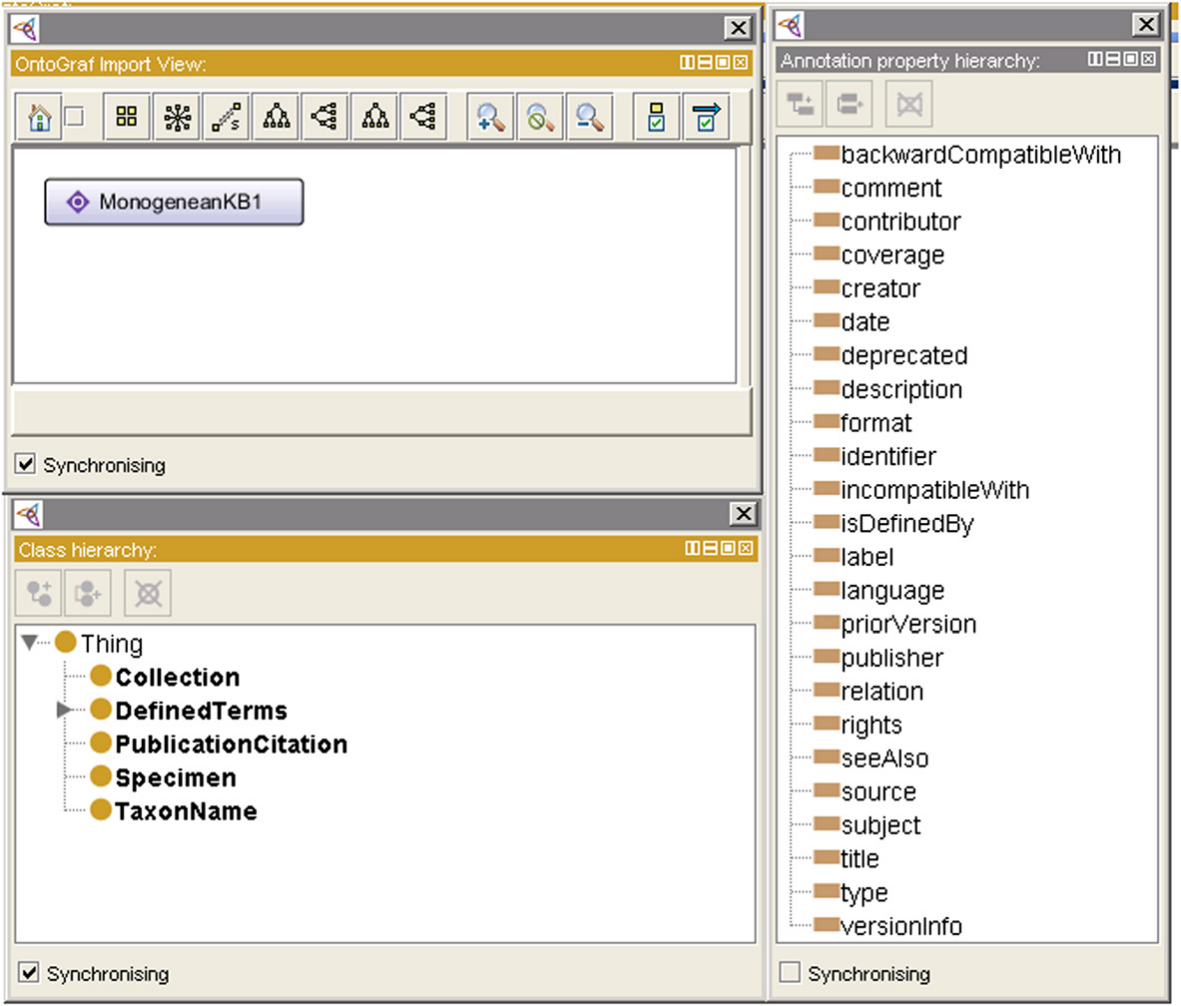
**Results of the Clarity criteria test (Test 3 and Test 7).** Visualization of MHBI ontology in Protégé. It shows that we have not imported any other system ontologies into the MHBI ontology, no classes in enumeration and no super or sub properties of Annotation properties.

As for Test 2 result, as presented in Figure 2, it is clearly show that no classes or properties in enumeration. Furthermore, for the Test 3 as illustrated in Figure 5, even though we have followed TDWG LSID standard for the vocabulary, we have created our own ontology based on our requirement study. Thus, we have not imported any other system ontologies. For the Test 7 result, we just used the built in Annotation property in Protégé [17] and there are no super or sub properties of Annotation properties as shown in Figure 5.

**Figure 6:**
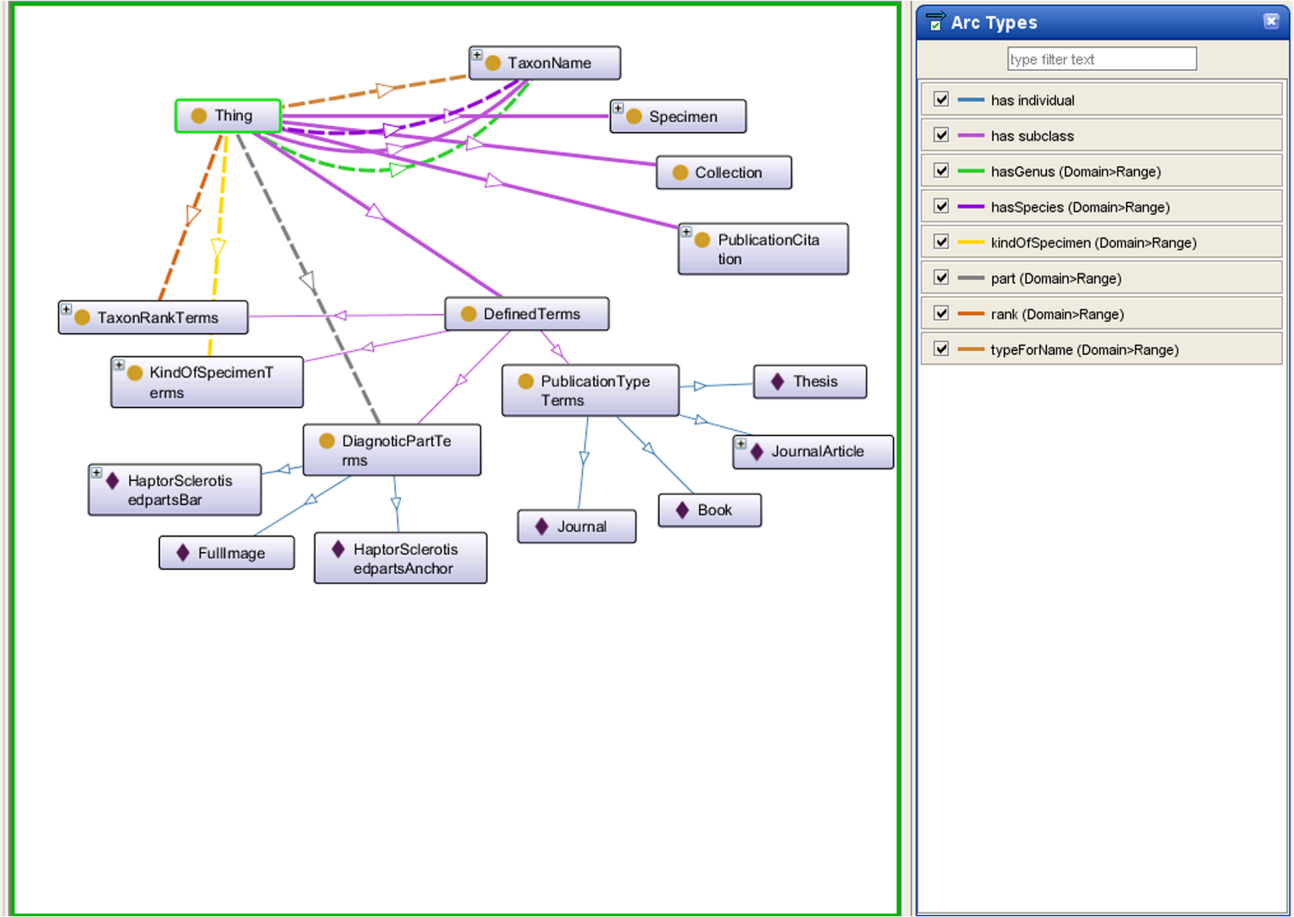
**MHBI ontology in a graph format.** Visualization of MHBI ontology using OntoGraf (Ontology Graph).

For Test 4, Test 5 and Test 6 results, as illustrated in Figure 6, in the MHBI ontology, there is no Meta-class, properties with class as range and sub classes of RDF classes.

#### Coherence

The definitions of concepts given in the ontology should be consistent. Only inferences consistent with existing definitions should be allowed. The formal part of the MHBI Ontology is checked by running the 12 consistency tests listed below and ensuring that, for these tests, all return true:

1. Domain of a Property should not be empty

2. Domain of a Property should not contain redundant Classes

3. Range of a Property should not contain redundant Classes

4. Domain of a Sub Property can only narrow Super Property

5. Range of a Sub Property can only narrow Super Property

6. Inverse of Functional must be Inverse Functional

7. Inverse of Inverse Functional must be Functional

8. Inverse of Sub Property must be Subproperty of Inverse of Super Property

9. Inverse of Symmetric Property must be Symmetric Property

10. Inverse of Top Level Property must be Top Level Property

11. Inverse of Transitive Property must be Transitive Property

12. Inverse Property must have matching Range and Domain

Results of the Test 1 to Test 3 are presented in Additional file 1. As shown in the results, domain and range of all the properties are assigned and no contain redundant classes.

**Figure 7:**
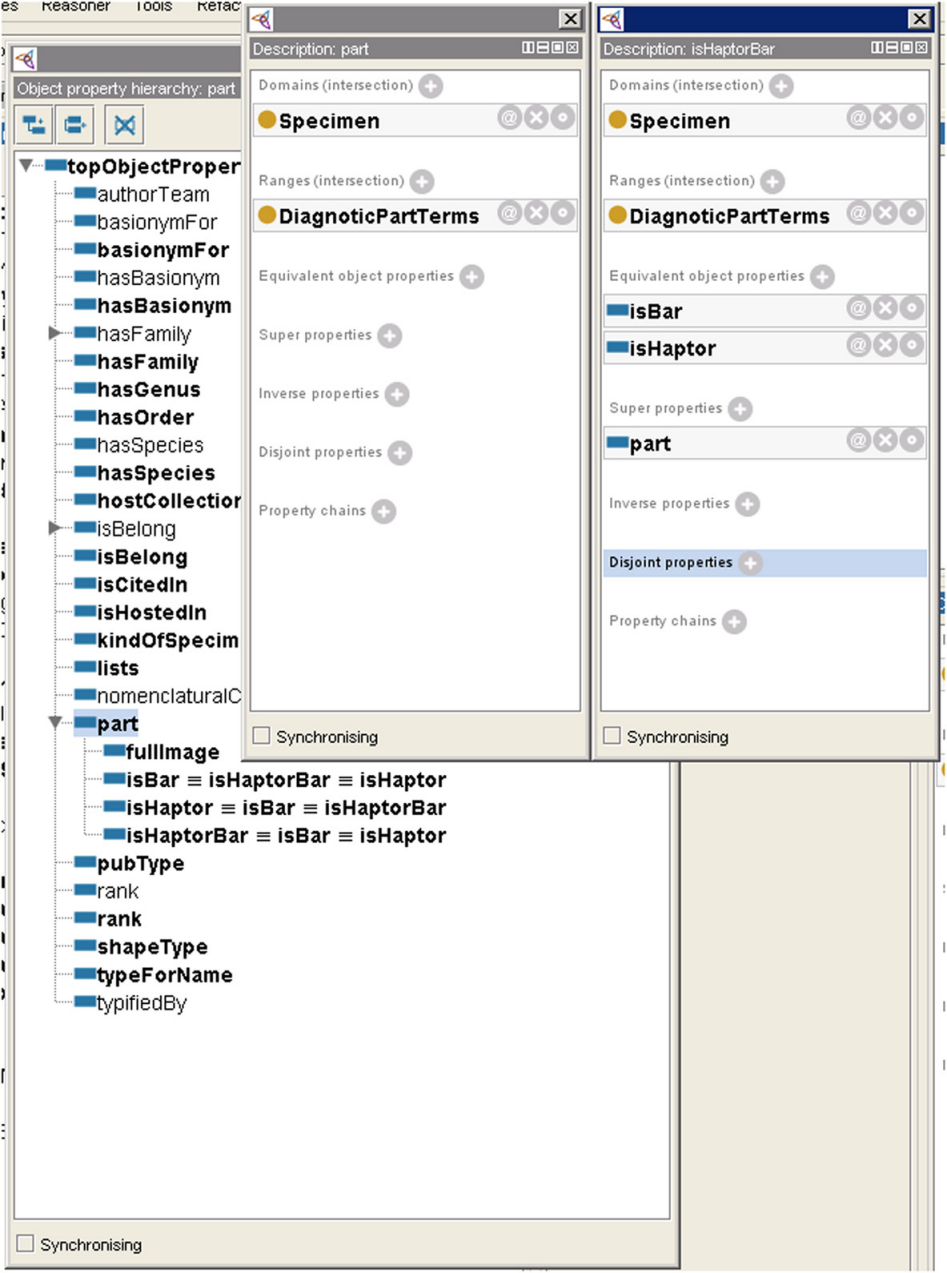
**Results of the Coherence criteria evaluation (Test 4, Test 5, Test 8 and Test 10).** In the MHBI ontology, *fullImage*, *isBar*, *isHaptor and isHaptorBar* are the sub properties of the *part* super property. Thus, domain and range of the sub property are defined by the super property.

The result of Test 4, Test 5, Test 8 and Test 10, are as illustrated in Figure 7. *ishaptorbar* property is a sub property of super property named *part*. Thus, domain and range of the sub property are defined by the super property. In this ontology, the *fullImage*, *isBar*, *isHaptor* and *isHaptorBar* sub properties are classified under *part* property. This is because, each specimen of haptoral bar image may annotate to any of these properties.

One of the results for Test 6 and Test 7 were applicable on the *typeForName* and *part* properties. If a property is inverse functional, then it means that the inverse property is functional [17]. For example, as illustrated in Figure 4, in this ontology, *typeForName* is functional property while *part* is inverse functional property. Thus, we can state that BifBaungi *typeForName* for bif-baungi-vb-i1, and then because of the inverse property we can infer that bif-baungi-vb-i1 *part* of BifBaungi.

An example for the result of Test 11 is illustrated as well in Figure 4. It shows an example of the transitive property *isBelong*. Since Bifbaungi *isbelong* to Bifurcohaptor, and Bifurcohaptor *isbelong* to Ancylodicoididae, then we can infer that Bifbaungi *isbelong* to Ancylodicoididae. As for inverse of transitive property *hasSpecies*, we can infer that Ancylodicoididae *hasSpecies* Bifbaungi. Furthermore, as presented in Additional file 1, inverse property in this example was fulfilled the Test 12 whereby it matched the range and domain.

**Figure 8:**
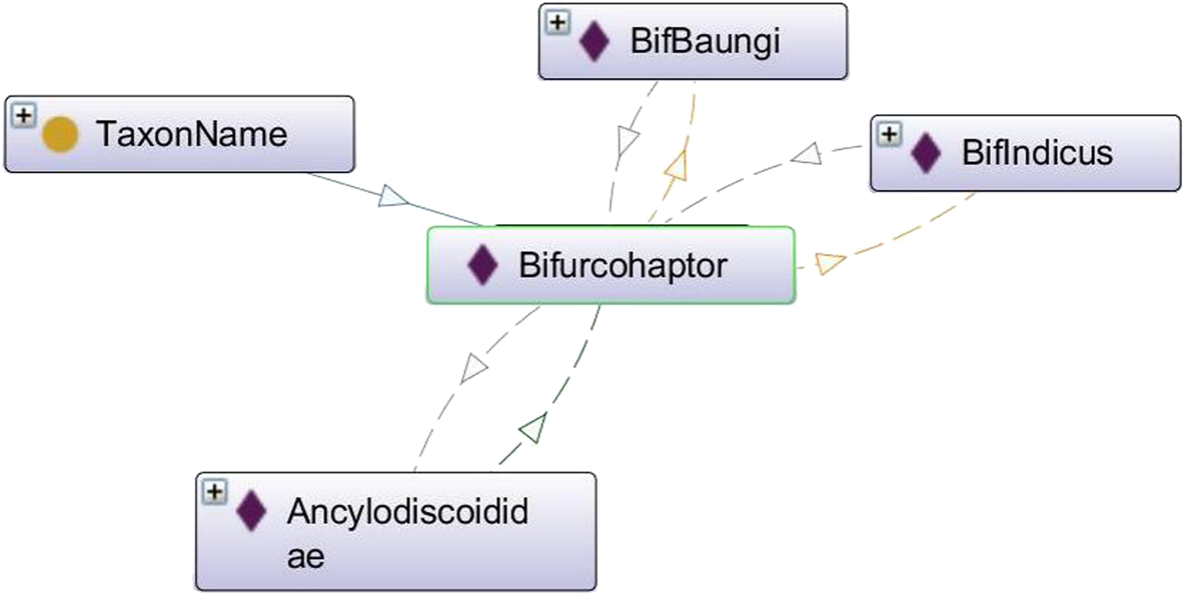
**Results of the Coherence criteria evaluation (Test 9).** An example of Symmetric property named *hasSynonym.* The instance BycGharui is related to the instance SiloGharui via the *hasSynonym* property. Then we can infer that SiloGharui must also be related to BycGharui via the *hasSynonym* property. Put another way, the property is its own inverse property.

Figure 8 illustrates an example of a Test 9 result. It shows an example of the symmetric property *hasSynonym*. The instance BycGharui is related to the instance SiloGharui via the *hasSynonym* property. Then we can infer that SiloGharui must also be related to BycGharui via the *hasSynonym* property. Put another way, the property is its own inverse property.

#### Extendibility

It should be possible to extend the ontology without altering the existing definitions. The requirement of easy ontology extension is quite an important feature as new knowledge emerges each day and may need to be added to an already existing ontology. To make MHBI Ontology extendable, the design consists of a hierarchical classification of concepts represented as classes, from general to specific. In MHBI ontology the notions classification, reasoning, and consistency are applied by defining new concepts from defined generic concepts. The concepts derived from generic concepts are placed precisely into the class hierarchy of MHBI Ontology to completely represent information defining a specimen.

**Figure 9:**
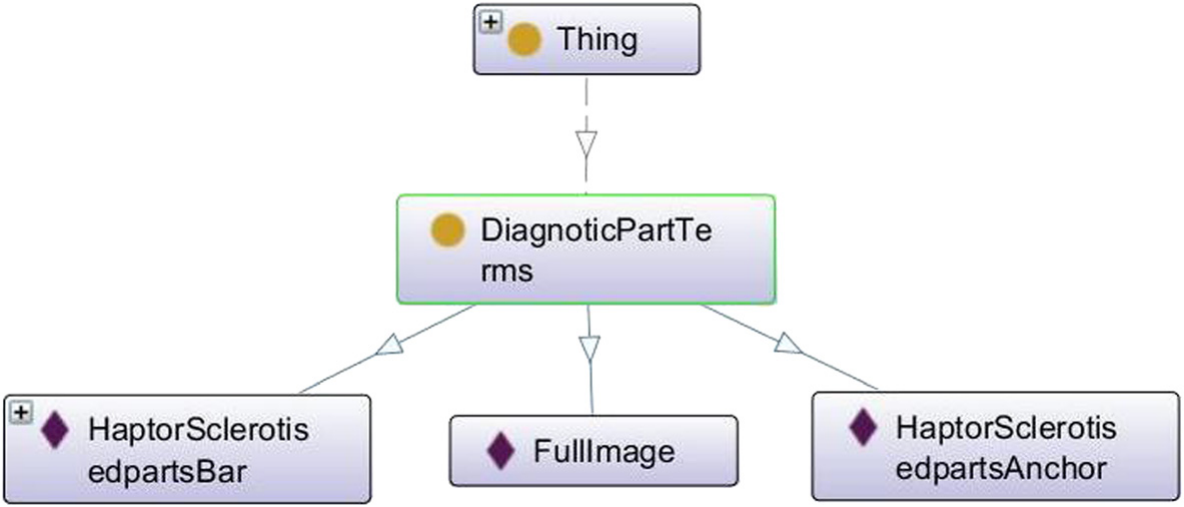
**Results of the Extendibility criteria evaluation.** In DiagnoticPartTerms concept of the MBHI ontology, we have considered the *HaptorSclerotisedpartBar*, *HaptorSclerotisedpartAnchor* and *FullImage*. In the future, we plan to add other diagnostic part such as *HaptorSclerotisedpartMarginalHook*, *HaptorSclerotisedpartPatch* and *HaptorSclerotisedpartOther*.

Figure 9 illustrates an example of this criterion. In the DiagnoticPartTerms concept of the MHBI ontology, we have considered *HaptorSclerotisedpartBar*, *HaptorSclerotisedpartAnchor* and *FullImage*. In the future we plan to include other diagnostic part such as *HaptorSclerotisedpartMarginalHook*, *HaptorSclerotisedpartPatch* and *HaptorSclerotisedpartOther*. Thus this ontology do not sanction a preference for one diagnostic part only and allow for the definition of other diagnostic parts, and a way to relate them to existing diagnostic parts.

#### Ontology commitment

**Figure 10:**
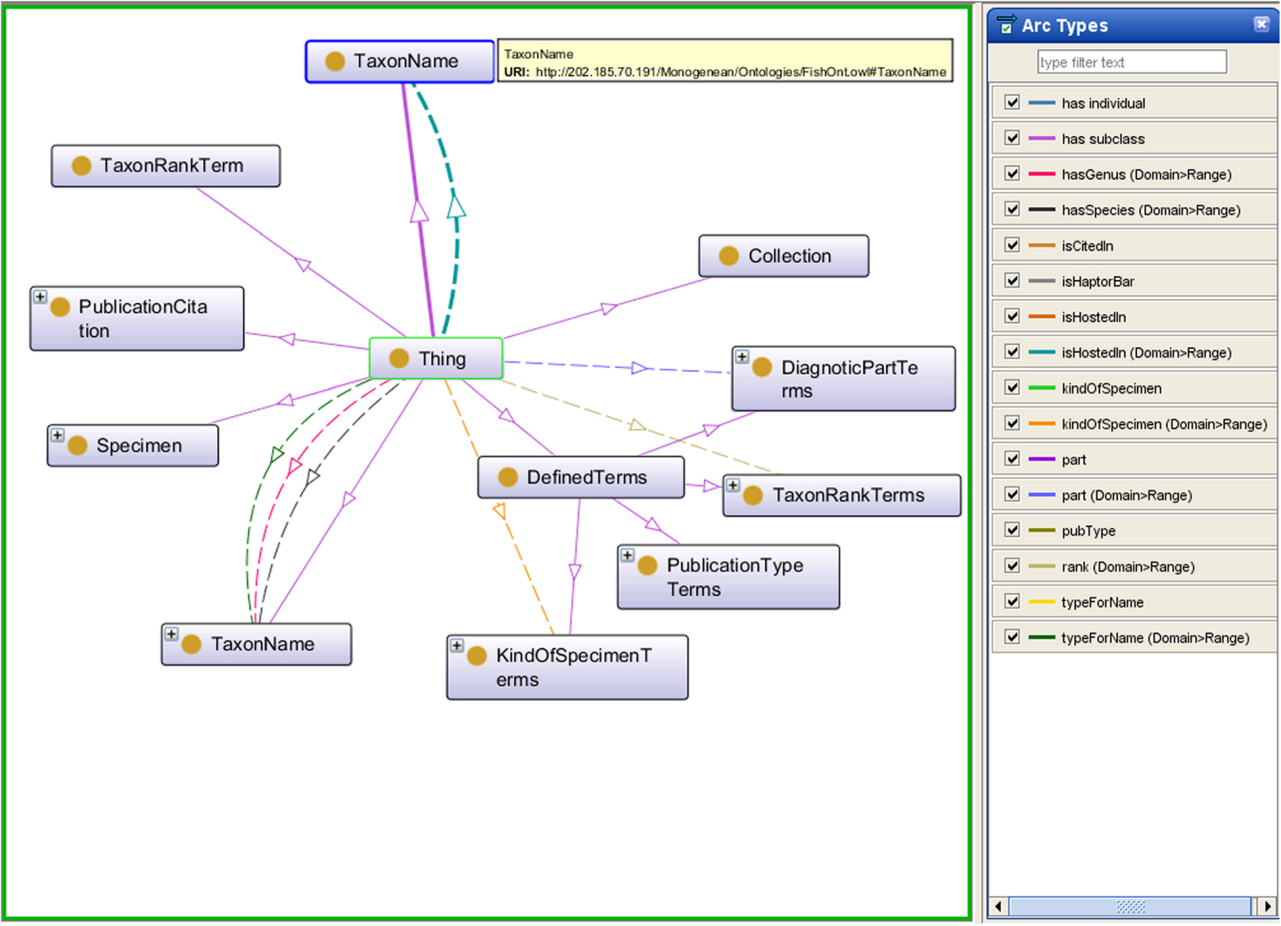
**Merged MHBI-Fish ontologies in a graph format.** Visualization of Merged MHBI-Fish ontologies using OntoGraf (Ontology Graph).

Ontology should make as few claims as possible about the domain while still supporting the intended knowledge sharing. MHBI Ontology will have as low an ontology commitment as domain ontology, because it reuses most of the concepts that have already been used to represent monogenean data and knowledge, and propose fewer new concepts. The low ontology commitment of the MHBI Ontology makes it more extendible and reusable as shown in Figure 10. Also, if fewer new concepts need to be agreed upon by the community, then this makes agreement easier.

#### Encoding bias

Ontology representation language should be as independent as possible from the use of the ontology. While developing MHBI Ontology, the choice of representation language as OWL [18] will keep the encoding bias to a minimum as MHBI ontology will be used by all stakeholders of taxonomy domain like: domain experts, pharmaceutical companies, researchers and students.

### Vocabularies

In this paper, we have used the TDWG LSID vocabulary to represent our data using semantics and we have also defined new vocabulary which is specific for annotating monogenean haptoral bar images (see Additional file 1 for the list and description).

### MHBI and MHBI-fish ontologies

We have developed a MHBI ontology as well as a merged MHBI-Fish ontologies. These ontologies can be viewed in a graph format (Figures 6 and 10).

## Discussion and conclusions

Semantic annotations of morphological descriptions that have been proposed till date have no information on the actual annotation of morphological descriptions or morphological images [19]. In this paper, we have annotated the monogenean images semantically and have developed a MHBI ontology which was eventually merged with a Fish ontology forming MHBI-Fish ontologies. This will enable linking of information from the monogenean ontology to the host species found in the fish ontology without changing the underlying schema for either of the ontologies.

To semantically represent our data we have used the vocabularies in TDWG LSID [9] which is the standard semantic naming convention for biodiversity information. We have also defined new vocabulary (Additional file 1) because this is the first time that images of the monogenean diagnostic hard part are being annotated semantically. In this paper, we have identified 7 concepts, and 27 properties (object and datatype properties in ontology) to represent descriptions of 159 images (*instance*s) (see Table 2).

In the future, we intend to work on developing a semantic query model through which a researcher can search using any word or phrase related to monogeneans and their hosts. In the future we also intend annotate images of other diagnostic hard parts to build a complete monogenean ontology. We will also build specific ontologies for the all the monogenean hosts such as fish, amphibians and reptiles. These ontologies will form the basis of a monogenean knowledge base to assist researchers in retrieving information for their analysis.

Furthermore, query results from the MHBI ontology presented in this paper are used as training set images for the Content Based Image Retrieval (CBIR). We have used this ontology to improve the efficiency of CBIR for Biodiversity [20,21]. As a result the relevancy rate of results provided by CBIR increases due to the decrease in the size of the training set as most the images are relevant to the query image. Also the retrieved images in the CBIR results are annotated, providing more information to the researcher.

## Competing interests

The authors declare that they have no competing interest.

## Authors’ contributions

SKD headed the study and structured the whole research. AA developed the system as part of her PhD. AS assisted in ontology evaluation. AA, LLHS and AS assisted in manuscript writing. All authors contributed in this study. All authors read and approved the final manuscript.

## Supplementary Material

Additional file 1 **LSID TDWG and new vocabularies.** LSID TDWG and new vocabularies (highlighted with gray background). The range of the vocabulary refers to the type of values for the object and datatype properties [10]. Click here for file
