# Supplementary material for: Semantic representation of monogenean haptoral Bar image annotation
Source: BMC Bioinformatics. 2013 Feb 12;14:48. doi: 10.1186/1471-2105-14-48 (PMC3639807; doi:10.1186/1471-2105-14-48)
Supplement: Additional file 1 — LSID TDWG and new vocabularies. LSID TDWG and new vocabularies (highlighted with gray background). The range of the vocabulary refers to the type of values for the object and datatype properties [10]. [file 1471-2105-14-48-S1.pdf]

Additional file 1

Vocabulary	Domain	Range	Description
**Concept**			
Specimen	-	-	It represents the record of specimen. The specimen includes image, fossil, herbarium, text or video. In this study it represents the illustrated images of the haptoral bars of the monogeneans
TaxonName	-	-	It represents a single scientific name
PublicationCitation	-	-	It represents a reference to a publication
DiagnosticPartTerms	-	-	It represents the name of the monogenean hard parts
KindOfSpecimenTerm	-	-	It represents the specimen terms such as Illustration,Digital Object, Still Image
TaxonRankTerms	-	-	It represents the taxon rank terms such as Species, Genus, Family, Order
PublicationTypeTerms	-	-	It represents the publication types such as Article, Journal, Book
**Object properties**			
kindOfSpecimen	Specimen	KindOfSpecimenTerms	The kind of object this specimen is e.g. Illustration, Digital Object, Still Image. It links to an instance of KindOfSpecimenTerms
part	Specimen	DiagnosticPartTerms	Which monogenean diagnostics hard part it represents
isHaptorBar	Specimen	DiagnosticPartTerms	The kind of diagnostic part this specimen is e.g. Haptor Sclerotised parts Bar, Haptor Sclerotised parts Anchor Full Image. It links to an instance of DiagnosticPartTerms
isCitedIn	Specimen	PublicationCitation	Where the specimen is cited in publication. It

			links to an instance of PublicationCitation
typeForName	TaxonName	TaxonName	A name for which this specimen is a type. It links to an instance of TaxonName
hasSynonym	TaxonName	TaxonName	The synonym of this name if it is a new name of the species
isHostedIn	Specimen	TaxonName	A link to the host species. It links to an instance of TaxonName in the merged monogenean image-fish ontology
rank	TaxonName	TaxonRankTerms	The taxonomic rank of this taxon e.g. Species, Genus, Family, and Order. It links to an instance of TaxonRankTerms
isBelong	TaxonName	TaxonName	Which taxon it belongs to. It links to an instance of TaxonName
hasSpecies	TaxonName	TaxonName	Species in the genus. It links to instances of TaxonName
hasGenus	TaxonName	TaxonName	Genus or genera in the Family. It links to instance(s) of TaxonName
hasFamily	TaxonName	TaxonName	Family or families in the Order. It links to instance(s) of TaxonName
hasOrder	TaxonName	TaxonName	Order or Orders in the Class. It links to instance(s) of TaxonName
pubType	PublicationTypeTerms	PublicationTypeTerms	The type of the publication e.g. Book, Journal Article, Journal. It links to an instance of PublicationTypeTerms
lists	TaxonName	TaxonName	Types of Taxon listed in the publication. It links to an instance of TaxonName
**Datatype properties**			
specimenId	Specimen	String	The museum deposition number of the specimen
imgDir	Specimen	String	The image path

			directory where the image is stored
imgDescription	Specimen	String	Description of the image
locality	TaxonName	String	Location where the specimen is collected
nameComplete	TaxonName	String	The complete name of the taxon
authorship	TaxonName	String	The name of all the authors to this taxon
year	TaxonName	String	The year of publication of this taxon
author	TaxonName	String	The authors of the publications
year	PublicationCitation	String	The year of the publication
title	PublicationCitation	String	The title of the publication
parentPublicationString	PublicationCitation	String	The name of journal of the publication.
number	PublicationCitation	String	The part number of the publication. E.g. 12, 325-330 means volume 12, p. 325-330
definedTerm		String	The complete name of the term
